# Dynamic Cloth Manipulation Considering Variable Stiffness and Material Change Using Deep Predictive Model With Parametric Bias

**DOI:** 10.3389/fnbot.2022.890695

**Published:** 2022-05-23

**Authors:** Kento Kawaharazuka, Akihiro Miki, Masahiro Bando, Kei Okada, Masayuki Inaba

**Affiliations:** JSK Robotics Laboratory, Department of Mechano-Informatics, Graduate School of Information Science and Technology, The University of Tokyo, Tokyo, Japan

**Keywords:** deep learning, predictive model, cloth manipulation, variable stiffness, parametric bias, musculoskeletal humanoid

## Abstract

Dynamic manipulation of flexible objects such as fabric, which is difficult to modelize, is one of the major challenges in robotics. With the development of deep learning, we are beginning to see results in simulations and some actual robots, but there are still many problems that have not yet been tackled. Humans can move their arms at high speed using their flexible bodies skillfully, and even when the material to be manipulated changes, they can manipulate the material after moving it several times and understanding its characteristics. Therefore, in this research, we focus on the following two points: (1) body control using a variable stiffness mechanism for more dynamic manipulation, and (2) response to changes in the material of the manipulated object using parametric bias. By incorporating these two approaches into a deep predictive model, we show through simulation and actual robot experiments that Musashi-W, a musculoskeletal humanoid with a variable stiffness mechanism, can dynamically manipulate cloth while detecting changes in the physical properties of the manipulated object.

## 1. Introduction

Manipulation of flexible objects such as fabric, which is difficult to modelize, is one of the major challenges in robotics. There are two main types of manipulation of flexible objects: static manipulation and dynamic manipulation. For each type, various model-based and learning-based methods have been developed. Static manipulation has been tackled for a long time, and various model-based methods exist (Inaba and Inoue, 1987; Saha and Isto, 2007; Elbrechter et al., 2012). Learning-based methods have also been actively pursued in recent years and have been successfully applied to actual robots (Lee et al., 2015; Tanaka et al., 2018). On the other hand, there are not so many examples of dynamic manipulation, which is more difficult compared to static manipulation. As for model-based methods, the study of Yamakawa et al. on cloth folding and knotting is well known (Yamakawa et al., 2010, 2011). Learning-based methods include Kawaharazuka et al. (2019b), which uses deep predictive models, and Jangir et al. (2020), which uses reinforcement learning. Also, there are many examples where only static manipulation is performed even though dynamic models are used (Ebert et al., 2018; Hoque et al., 2020). There is an example where the primitive of the dynamic motion is generated manually and only the grasping point is trained (Ha and Song, 2021).

**Figure 1:**
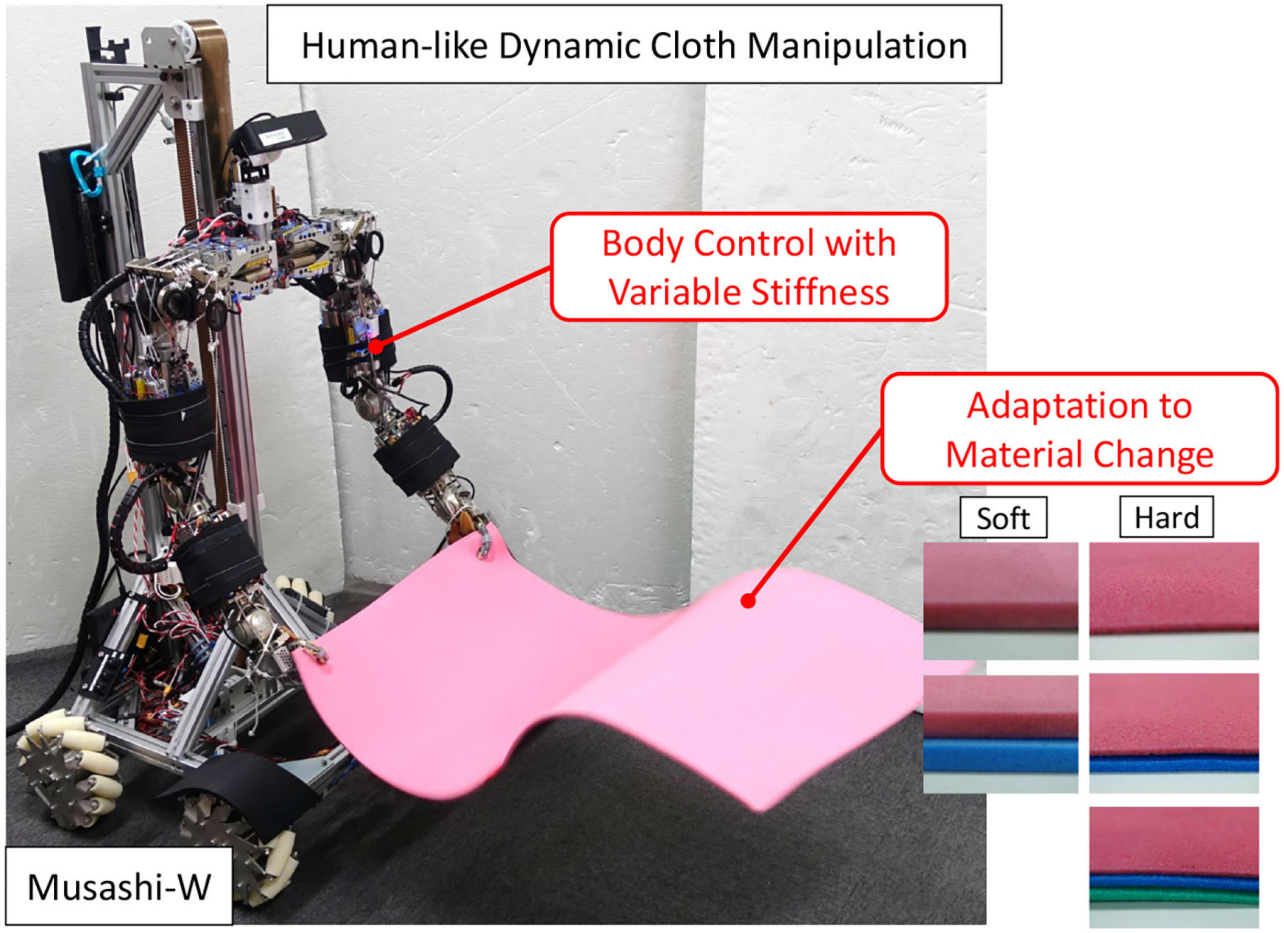
Dynamic cloth manipulation by the musculoskeletal humanoid Musashi-W considering body control with variable stiffness and adaptation to material change.

In this study, we handle dynamic manipulation such as the spreading of a bed sheet or a picnic sheet. For the dynamic manipulation, there are several points lacking in human-like adaptive dynamic cloth manipulation that have not been addressed in the introduced previous studies. The previous studies have not been able to utilize the flexible bodies of robots to move their arms at high speed as humans do, and to perform manipulation based on an understanding of the characteristics of the material from a few trials, even if the material to be manipulated changes. Therefore, we focus on two points: (1) body control for more dynamic manipulation, and (2) adaptation to changes in the material of the manipulated object (Figure 1). In (1), we aim at cloth manipulation by a robot with a variable stiffness mechanism, which is flexible like a human and can manipulate its flexibility at will. In this study, we perform manipulation by Musashi-W (MusashiDarm Kawaharazuka et al., 2019a with wheeled base), a musculoskeletal humanoid with variable stiffness mechanism using redundant muscles and nonlinear elastic elements. We consider how the dynamic cloth manipulation is changed by using the additional stiffness value as the control command of the body. There have been examples of dynamic pitching behavior by changing hardware stiffness in the framework of model-based optimal control (Braun et al., 2012), and several static tasks by changing software impedance using reinforcement learning (Martín-Martín et al., 2019). On the other hand, there is no example focusing on changes in the hardware stiffness in a dynamic handling task of difficult-to-modelize objects that require learning-based control, such as in this study. In (2), we aim at adapting to changes in the material of the manipulated object using parametric bias (Tani, 2002). The parametric bias is an additional bias term in neural networks, which has been mainly used for imitation learning to extract multiple attractor dynamics from various motion data (Ogata et al., 2005; Kawaharazuka et al., 2021). In this study, we use it to embed information about the material and physical properties of the cloth. When the robot holds a new cloth, the physical properties can be identified by manipulating the cloth several times, and dynamic cloth manipulation can be accurately performed. Parametric bias can be applied not only to simulations but also to actual robots where it is difficult to identify the parameters of cloth materials since it implicitly self-organizes from differences in dynamics of each data. In this study, we construct a deep predictive model that incorporates (1) and (2) and demonstrate the effectiveness by performing a more human-like dynamic cloth manipulation.

The contribution of this study is as follows.

- Examination of the effect of adding the body stiffness value to the control command.
- Adaptation to changes in cloth material using parametric bias.
- Human-like adaptive dynamic cloth manipulation by learning a deep predictive model considering variable stiffness and material change.

In Section 2, we describe the structure of the deep predictive model, the details of variable stiffness control, the estimation of material parameters, and the body control for dynamic cloth manipulation. In Section 3, we discuss online learning of material parameters and changes in dynamic cloth manipulation due to variable stiffness and cloth material changes in simulation and the actual robot. In addition, a table setting experiment including dynamic cloth manipulation is conducted. The results of the experiments are discussed in Section 4, and the conclusion is given in Section 5.

## 2. Dynamic Cloth Manipulation Considering Variable Stiffness and Material Change

**Figure 2:**
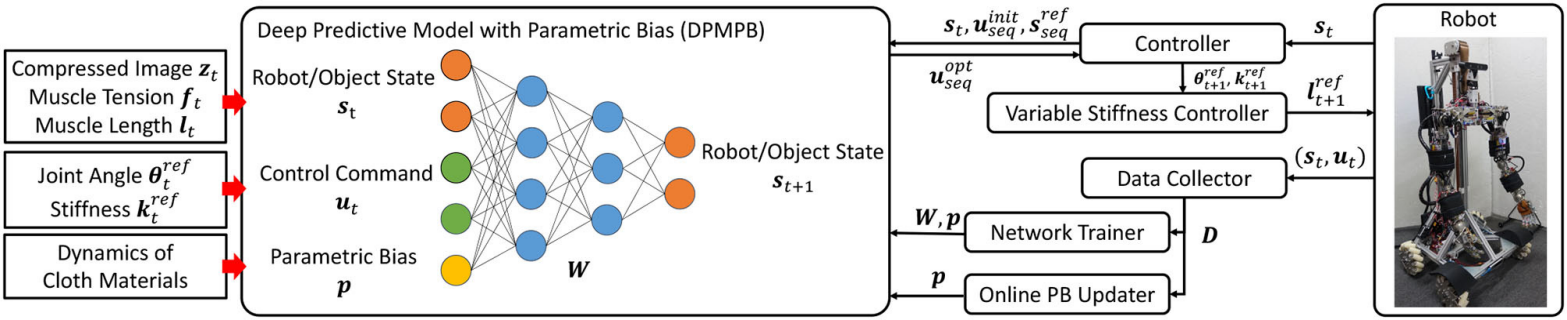
The overview of our system: deep predictive model with parametric bias (DPMPB), controller using DPMPB for dynamic cloth manipulation, variable stiffness controller for musculoskeletal humanoids, data collector for DPMPB, and online updater of parametric bias (PB).

We call the network used in this study Dynamic Predictive Model with Parametric Bias (DPMPB). The entire system is shown in Figure 2.

### 2.1. Network Structure of DPMPB

Dynamic Predictive Model with Parametric Bias can be expressed by the following equation.


(1) $$\begin{array}{l} s_{t+1}=h_{dpmpb}\left(s_{t},u,p\right) \end{array}$$

where *t* is the current time step, ***s*** is the state of the manipulated object and robot, ***u*** is the control command to the robot body, ***p*** is the parametric bias (PB), and ***h***_*dpmpb*_ is the function representing the time series change in the state of the manipulated object and robot due to the control command. We use the state of the cloth and the state of the robot for ***s*** in this study of cloth manipulation. For the cloth state, we use ***z***_*t*_, which is the compressed value of the current image *I*_*t*_ by using AutoEncoder (Hinton and Salakhutdinov, 2006). Although the robot state should be different for each robot, we use ***f***_*t*_ and ***l***_*t*_ for the musculoskeletal humanoid used in this study (***f***_*t*_ and ***l***_*t*_ represent the current muscle tension and muscle length). Thus, $s_{t}^{T}=\left(\begin{array}{ccc} z_{t}^{T} & f_{t}^{T} & l_{t}^{T} \end{array}\right)$. Also, we set $u^{T}=\left(\begin{array}{cc} \theta^{ref,T} & k^{ref,T} \end{array}\right)$ (***θ***^*ref*^ represents the target joint angle and ***k***^*ref*^ represents the target body stiffness value). The details of the target joint angle and body stiffness are described in Section 2.2. The parametric bias is a value that can embed implicit differences in dynamics, which are common for the same material and different for different materials. By collecting data using various cloth materials, information on the dynamics of the material of the manipulated object is embedded in ***p***. Note that each sensor value is used as input to the network after normalization using all the obtained data.

In this study, the DPMPB consists of 10 layers: 4 fully-connected layers, 2 LSTM layers (Hochreiter and Schmidhuber, 1997), and 4 fully-connected layers, in order. The number of units is set to {*N*_*u*_ + *N*_*s*_ + *N*_*p*_, 300, 100, 30, 30 (number of units in LSTM), 30 (number of units in LSTM), 30, 100, 300, *N*_*s*_} (where *N*_{*u, s, p*}_ is the number of dimensions of {***u***, ***s***, ***p***}). The activation function is hyperbolic tangent and the update rule is Adam (Kingma and Ba, 2015). Regarding the AutoEncoder, the image is compressed by applying convolutional layers with kernel size 3 and stride 2 five times to a 128 × 96 binary image (the cloth part is extracted in color), then reducing the dimensionality to 256 and 3 units, in order, using the fully-connected layers, and finally restoring the image by the fully-connected layers and the deconvolutional layers. For all layers exempting the last layer, batch normalization (Ioffe and Szegedy, 2015) is applied, and the activation function is ReLU (Nair and Hinton, 2010). The dimension of ***p*** is set to 2 in this study, which should be sufficiently smaller than the number of cloth materials used for training. The execution period of Equation (1) is set to 5 Hz.

### 2.2. Variable Stiffness Control for Musculoskeletal Humanoids

Regarding the musculoskeletal humanoid with variable stiffness mechanism used in this study, we will describe the details of ***θ***^*ref*^ and ***k***^*ref*^. In this study, we use the following relationship of joint angle ***θ***, muscle tension ***f***, and muscle length ***l*** (Kawaharazuka et al., 2018).


(2) $$\begin{array}{l} l=h_{bodyimage}\left(\theta ,f\right) \end{array}$$

***h***_*bodyimage*_ is trained using the actual robot sensor information. When using this trained network for control, the target joint angle ***θ***^*ref*^ and the target muscle tension ***f***^*ref*^ are determined and the corresponding target muscle length $l^{ref}=h_{bodyimage}\left(\theta^{ref},f^{ref}\right)$ is calculated. However, since this value is the muscle length to be measured, not the target muscle length, ***l***^*send*^, which takes into account the muscle elongation in muscle stiffness control (Shirai et al., 2011), is sent to the actual robot (Kawaharazuka et al., 2018) in practice.

Here, we consider how ***f***^*ref*^ is given. Normally, ***f***^*ref*^ should be the value required to realize ***θ***^*ref*^. On the other hand, in Kawaharazuka et al. (2018), a constant value *f*^*const*^ is first given to ***f***^*ref*^ for all muscles to achieve ***θ***^*ref*^ to a certain degree, and then the current muscle tension ***f*** is given as ***f***^*ref*^ to achieve ***θ***^*ref*^ more accurately. The body stiffness can be changed to some extent depending on the value of *f*^*const*^ sent at the beginning. In addition, since the relationship between ***s*** and ***u*** is eventually acquired by learning in this study, it is not necessary to realize ***θ***^*ref*^ precisely. Therefore, we use *f*^*const*^ as the body stiffness value ***k***^*ref*^ included in ***u*** and operate the robot with $l^{ref}=h_{bodyimage}\left(\theta^{ref},f^{const}\right)$. By acquiring training data while changing *f*^*const*^, we can perform dynamic cloth manipulation considering the body stiffness value.

**Figure 3:**
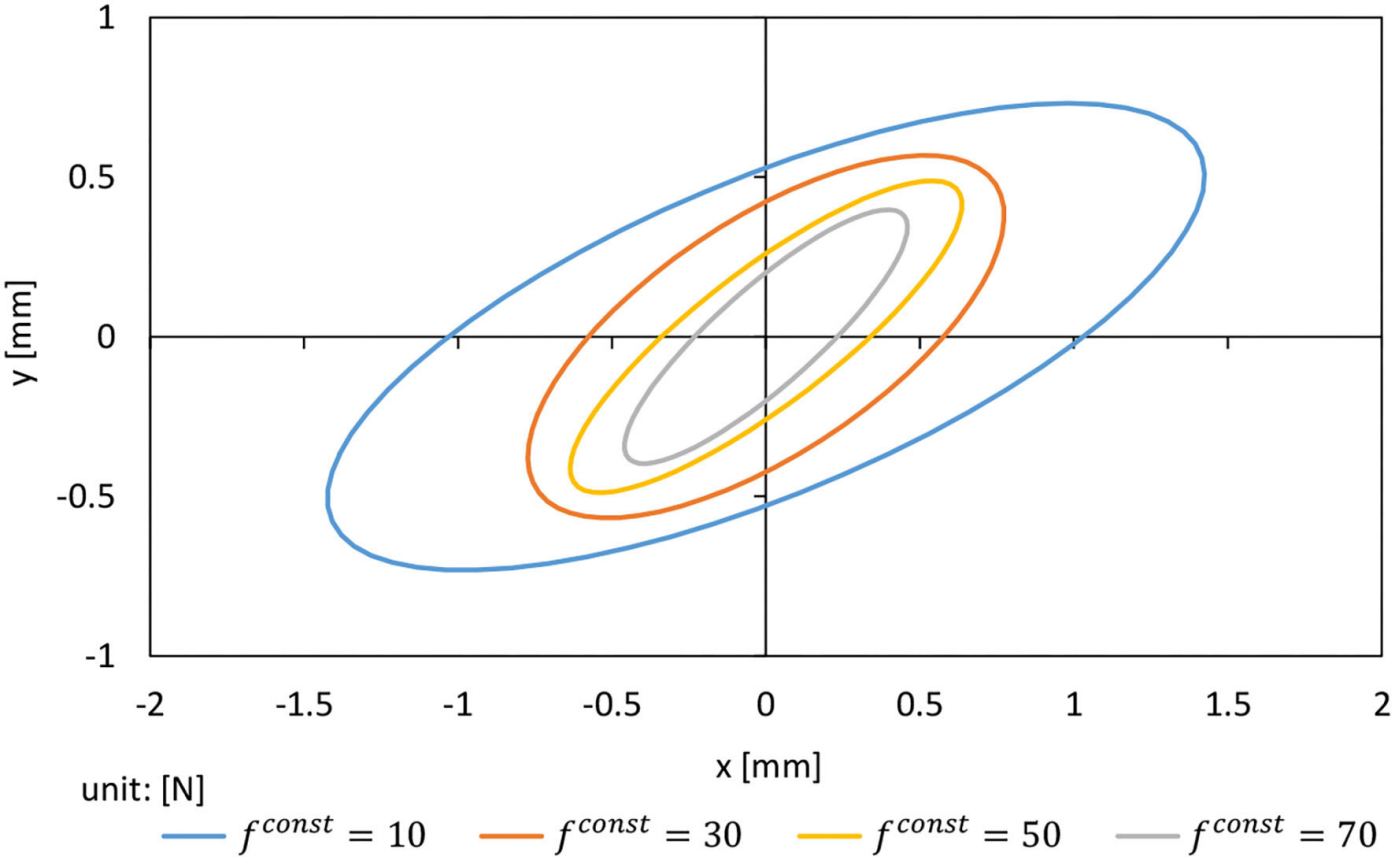
Operational stiffness ellipsoid when adding force of 1 N while changing *f*^*const*^ = {10, 30, 50, 70} [N].

In the following, we show the results of experiments to see how much the operational stiffness of the arm changes with *f*^*const*^. Figure 3 shows the operational stiffness ellipsoid of the left arm in the sagittal plane when ***θ***^*ref*^ is set as the state with the elbow of the left arm of Musashi-W bent at 90 degrees and *f*^*const*^ is changed to {10, 30, 50, 70} [N]. The graph shows the displacement of the hand when a force of 1 N is applied from all directions. It can be seen that the size of the stiffness ellipsoid changes greatly with the change in *f*^*const*^. Therefore, we use *f*^*const*^ as the body stiffness value ***k***^*ref*^ in this study.

### 2.3. Training of DPMPB

By setting ***u*** randomly, or operating the robot with GUI or VR device, we collect the data of ***s*** and ***u***. For a single, coherent manipulation trial *k*, performed with the same cloth, the data *D*_*k*_ = {(***s***_1_, ***u***_1_), (***s***_2_, ***u***_2_), ⋯ , (***s***_*T*_*k*__, ***u***_*T*_*k*__)} (1 ≤ *k* ≤ *K*, where *K* is the total number of trials and *T*_*k*_ is the number of time steps for the trial *k*). Then, we obtain the data *D*_*train*_ = {(*D*_1_, ***p***_1_), (*D*_2_, ***p***_2_), ⋯ , (*D*_*K*_, ***p***_*K*_)} for training. ***p***_*k*_ is the parametric bias for the trial *k*, a variable that has a common value during that one trial and a different value for different trials. We use this data *D*_*train*_ to train the DPMPB. In the usual training, only the network weight *W* is updated, but in this study, *W* and ***p***_*k*_ are updated simultaneously as below,


(3) $$\begin{array}{l} W\leftarrow W-\beta \frac{\partial L}{\partial W} \end{array}$$

(4) $$\begin{array}{l} p_{k}\leftarrow p_{k}-\beta \frac{\partial L}{\partial p_{k}} \end{array}$$

where *L* is the loss function (mean squared error, in this study) and β is the learning rate. ***p***_*k*_ will be embedded with the difference in dynamics in each trial, i.e., the dynamics of the manipulated cloth in this study. ***p***_*k*_ is trained with an initial value of **0**, and it is not necessary to directly provide parameters related to the dynamics of the cloth. Therefore, parametric bias can be applied to cases where the dynamics parameters are not known, as in the handling of actual cloth.

### 2.4. Online Estimation of Cloth Material

When manipulating a new cloth, if we do not know its correct dynamics, we will not be able to perform the manipulation correctly. Therefore, we need to obtain data on how the shape of the cloth changes when the cloth is manipulated, and estimate the dynamics of the cloth, i.e., ***p*** in this study, based on this data. First, we obtain the data *D*_*new*_ (*D*_*train*_ for one trial with *K* = 1) as in Section 2.3 when the cloth is manipulated by random commands, GUI, or the controller described later (Section 2.5). Using this data, we update only ***p*** while *W* is fixed, unlike the training process of Section 2.3. We do not execute Equation (3) but update only ***p*** by Equation (4) using the current ***p*** as the initial value. Since ***p*** is of a very low dimension compared to *W*, the overfitting problem can be prevented. In other words, by updating only the terms related to the cloth dynamics, we make the dynamics of DPMPB consistent with *D*_*new*_. In this case, the update rule is MomentumSGD.

### 2.5. Dynamic Cloth Manipulation Using DPMPB

We describe the dynamic cloth manipulation control using DPMPB. First, we obtain the target image *I*^*ref*^, which is the target state of the cloth, and compress it to ***z***^*ref*^ by AutoEncoder. ***f***^*ref*^ and ***l***^*ref*^ are set to **0**, and ***s***^*ref*^ is generated by combining them. Next, we determine the number of expansions of DPMPB $N_{seq}^{control}$ and set the initial value $u_{seq}^{init}$ as $u_{seq}^{opt}$, which is $N_{seq}^{control}$ steps of ***u*** to be optimized (the abbreviation of $u_{\left[t,t+N_{seq}^{control}-1\right]}^{opt}$). We calculate the loss function as follows, and update $u_{seq}^{opt}$ from this value while *W* and ***p*** are fixed.


(5) $$\begin{array}{l} L=h_{loss}\left(s_{seq}^{ref},s_{seq}^{pred}\right) \end{array}$$

(6) $$\begin{array}{l} g=\frac{\partial L}{\partial u_{seq}^{opt}} \end{array}$$

(7) $$\begin{array}{l} u_{seq}^{opt}\leftarrow u_{seq}^{opt}-\gamma \frac{g}{||g{||}_{2}} \end{array}$$

where *h*_*loss*_ is the loss function (to be explained later), $s_{seq}^{ref}$ is a time series of the target cloth states with ***s***^*ref*^ arranged in $N_{seq}^{control}$ steps, $s_{seq}^{pred}$ is $s_{\left[t+1,t+N_{seq}^{control}\right]}^{pred}$, which is the time series of ***s*** predicted when $u_{seq}^{opt}$ is applied to the current state ***s***_*t*_, ||·||_2_ is L2 norm, and γ is the learning rate. Equation (6) can be computed by the backpropagation method of neural networks, and Equation (7) represents the gradient descent method. In other words, the time series control command is updated to make the predicted time series of ***s*** closer to the target value. Note that *W* is obtained in Section 2.3, and ***p*** is obtained in Section 2.4. Here, γ can be constant, but in this study, we update $u_{seq}^{opt}$ using multiple values of γ for faster convergence. We use the value obtained by dividing [0, γ_*max*_] into $N_{batch}^{control}$ equal logarithmic intervals (γ_*max*_ is the maximum value of γ) as γ, update $u_{seq}^{opt}$ using each γ, and adopt $u_{seq}^{opt}$ with the smallest *L* among them, repeating the process $N_{iter}^{control}$ times. An appropriate γ is selected for each iteration and Equation (7) is performed. Also, for the initial value $u_{seq}^{init}$ of $u_{seq}^{opt}$, we use the value optimized in the previous step $u_{seq}^{prev}$ (the abbreviation of $u_{\left[t,t+N_{seq}^{control}-1\right]}^{prev}$) by shifting it one step to the left and duplicating the last term, $u_{\left\{t+1,\cdots \;,t+N_{seq}^{control}-1,t+N_{seq}^{control}-1\right\}}^{prev}$. This allows us to achieve faster convergence, taking into account the previous optimization results. The final obtained target value $u_{t}^{opt}$ at the current time step of $u_{seq}^{opt}$ is sent to the actual robot.

Here, we consider dynamic cloth manipulation tasks such as spreading out a bed sheet or a picnic sheet in the air. Let ***s***^*ref*^ be the target value that contains the image ***z***^*ref*^ of the unfolded state of the cloth, and we consider trying to achieve the target value. In this case, if the cloth does not spread well at the first attempt, it would be not possible to make minor adjustments, and it is necessary to generate a series of motions to try to spread the cloth from the beginning. In other words, even if the loss is lowered to some extent by the first attempt, it is necessary to go through a state where the loss is increased by large motions in order to lower the loss further. Therefore, if we just define $s_{seq}^{ref}$ as a vector of the same ***s***^*ref*^, we end up with $u_{seq}^{opt}$ that hardly moves after the first attempt. The same problem occurs when ***s***^*ref*^ is defined as the state of spreading a cloth in the air since that state is not always realized. Thus, it is necessary to realize the target state by periodically spreading the cloth over and over again. Based on this feature, in this study, we determine the number of periodic steps of the periodic motion, $N_{periodic}^{control}$, and change *h*_*loss*_ for each period. In this study, *h*_*loss*_ is expressed as follows.


(8) $$\begin{array}{l} h_{loss}\left(s_{seq}^{ref},s_{seq}^{pred}\right)=||m_{t}⊗\left(z_{seq}^{ref}-z_{seq}^{pred}\right){||}_{2}+w_{loss}||f_{seq}^{pred}{||}_{2} \end{array}$$

where ${\left\{z,f\right\}}_{seq}^{\left\{ref,pred\right\}}$ is the value of {***z***, ***f***} extracted from $s_{seq}^{\left\{ref,pred\right\}}$, and *w*_*loss*_ is the weight constant. ***m***_*t*_ ($\in {\left\{0,1\right\}}^{N_{seq}^{control}}$) is a vector with 1 occurring at every $N_{periodic}^{control}$ step and 0, otherwise. It is shifted to the left at each time step, and 0 or 1 is inserted from the right depending on $N_{periodic}^{control}$ (e.g., if $N_{seq}^{control}=N_{periodic}^{control}=4$, then (0, 1, 0, 0) → (1, 0, 0, 0) → (0, 0, 0, 1) in this order). This makes it possible to bring the cloth state ***z*** closer to the target value every $N_{periodic}^{control}$ step and enables dynamic cloth manipulation that handles periodic states which can only be realized momentarily. The second term on the right-hand side of Equation (8) is a term to minimize muscle tension as much as possible.

In this study, we set $N_{seq}^{control}=8$, $N_{batch}^{control}=30$, γ_*max*_ = 1.0, $N_{iter}^{control}=3$, *w*_*loss*_ = 0.001, and $N_{periodic}^{control}=8$.

## 3. Experiments

### 3.1. Experimental Setup

**Figure 4:**
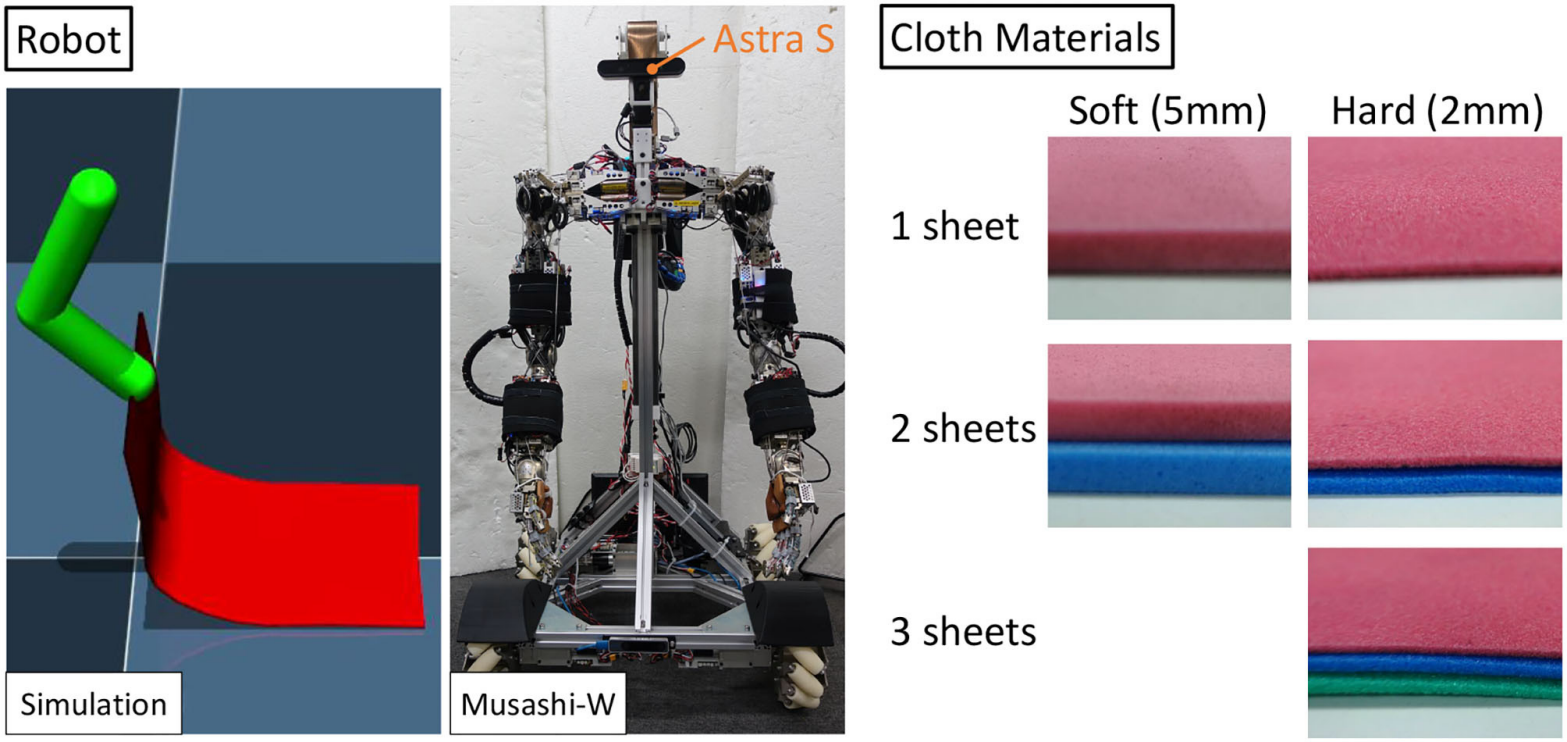
Experimental setup: the simulated simple robot and the musculoskeletal humanoid Musashi-W used in this study and cloth materials of soft and hard type polyethylene foam.

The robot and the types of cloth used in this study are shown in Figure 4. First, we conduct a cloth manipulation experiment using a simple 2-DOF robot in simulation with Mujoco (Todorov et al., 2012). Here, the cloth is modeled as a collection of 3 ×6 point masses, and the damping *C*_*damp*_ between those point masses and the weight *C*_*mass*_ of the entire cloth can be changed. Next, we use the actual robot Musashi-W, which is a musculoskeletal dual arm robot MusashiDarm (Kawaharazuka et al., 2019a) with a mechanum wheeled base and a z-axis slider. The head is equipped with Astra S camera (Orbbec 3D Technology International, Inc.). In this study, we use two kinds of cloth: soft type (5 mm thick) and hard type (2 mm thick) of polyethylene foam (TAKASHIMA color sheet, WAKI Factory, Inc.). Polyethylene foam was used instead of actual cloth in this study due to the joint speed limitation of Musashi-W. For the soft type, we prepare one or two sheets, and for the hard type, we prepare one, two, or three sheets (denoted as soft-1, soft-2, hard-1, hard-2, hard-3).

We move the arms of the simulated robot and Musashi-W only in the sagittal plane, with two degrees of freedom in the shoulder and elbow pitch axes. Although the control command of the simulated robot is two dimensional without hardware body stiffness, the control command of Musashi-W is three dimensional including the stiffness (hardware stiffness was difficult to reproduce in simulation). The image is compressed by AutoEncoder after color extraction, binarization, closing opening, and resizing (the front part of the cloth is red and the back part is a different color such as blue or black).

### 3.2. Simulation Experiment

**Figure 5:**
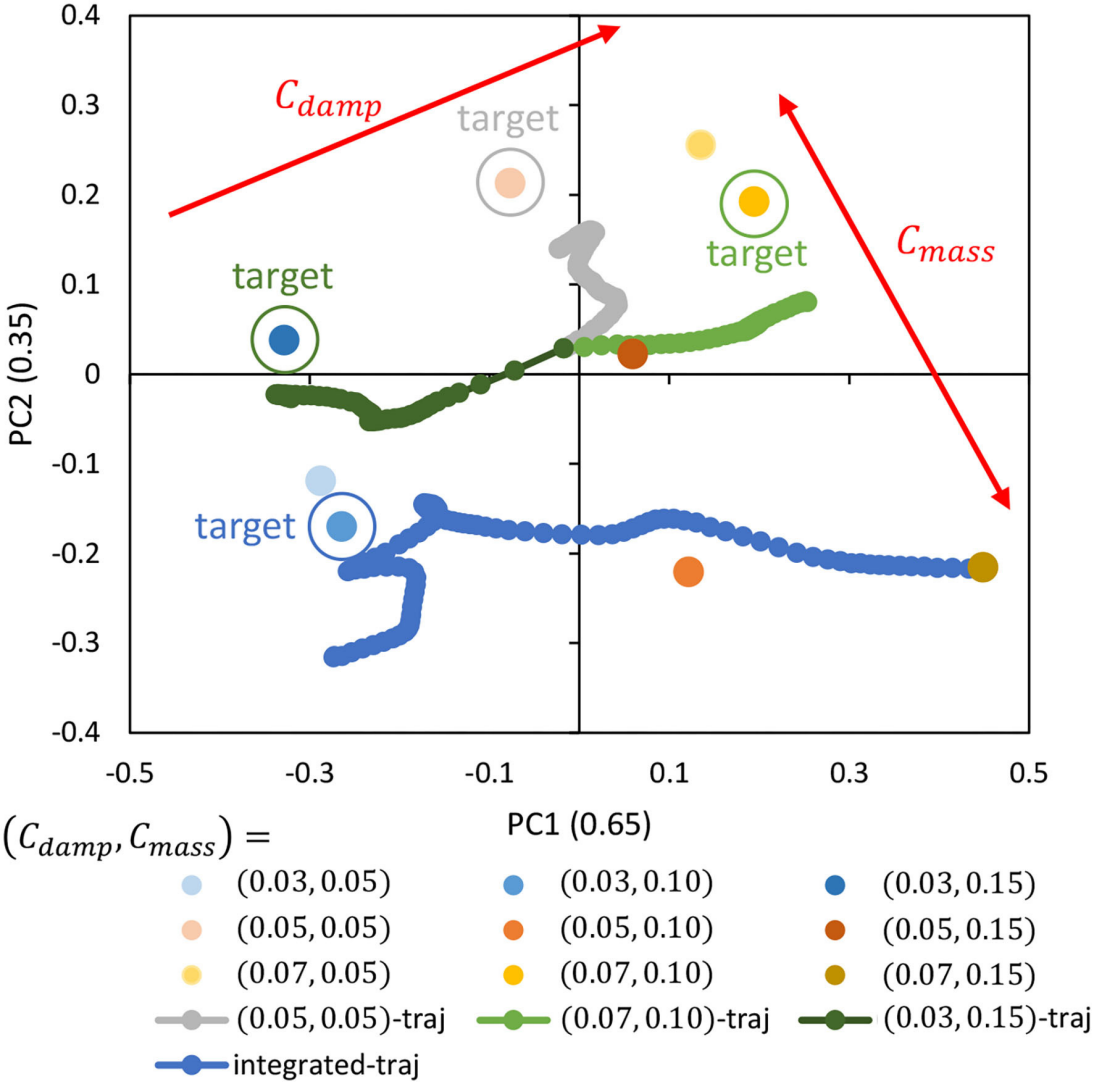
Simulation experiment: the trained parametric bias when setting *C*_*damp*_ = {0.03, 0.05, 0.07} and *C*_*mass*_ = {0.05, 0.10, 0.15}, the trajectory of online updated parametric bias when setting (*C*_*damp*_, *C*_*mass*_) = {(0.05, 0.05), (0.07, 0.10), (0.03, 0.15)}, and the trajectory of online updated parametric bias when setting (*C*_*damp*_, *C*_*mass*_) = (0.03, 0.10) for the integrated experiment.

The cloth characteristics are changed to *C*_*damp*_ = {0.03, 0.05, 0.07} and *C*_*mass*_ = {0.05, 0.10, 0.15} while randomly commanding ***θ***^*ref*^ (Random) for about 50 s, and while human operation by GUI (joint angle is given by GUI) for about 50 s. Using a total of 4,500 data points, we trained DPMPB with the number of LSTM expansions set to 30, the number of batches being 300, and the number of epochs being 300 (these parameters are empirically set). Principle Component Analysis (PCA) is applied to the parametric bias for each cloth obtained in the training, and its arrangement in the two-dimensional plane is shown in Figure 5. Note that even if ***p*** is two-dimensional, the axes of the principal components, PC1 and PC2, become more defined by applying PCA. It can be seen that the PBs are neatly arranged mainly on the axis of PC1 according to the magnitude of *C*_*damp*_. On the other hand, for the axis of PC2, some PBs are not arranged according to the magnitude of *C*_*mass*_, and we can see that the dynamics change due to *C*_*mass*_ is complex. Also, the contribution ratio in PCA is 0.65 for PC1 and 0.35 for PC2, indicating that the influence of *C*_*mass*_ on the dynamics of the cloth is small.

Here, with each cloth of (*C*_*damp*_, *C*_*mass*_) = {(0.05, 0.05), (0.07, 0.10), (0.03, 0.15)}, the Random motion and the online material estimation in Section 2.4 are executed for about 40 s. The trajectory of the parametric bias (traj) is shown in Figure 5. Note that the initial value of PB is **0** (the origin of PB is not necessarily at the origin of the figure, since PCA is applied). It can be seen that the current PB gradually approaches the respective PB values obtained during training in the dynamics of the current cloth. In particular, the accuracy of the online estimation is high for the axis of *C*_*damp*_. On the other hand, for the axis of *C*_*mass*_, though the material can be correctly recognized to some extent, the accuracy is less than for the axis of *C*_*damp*_.

**Figure 6:**
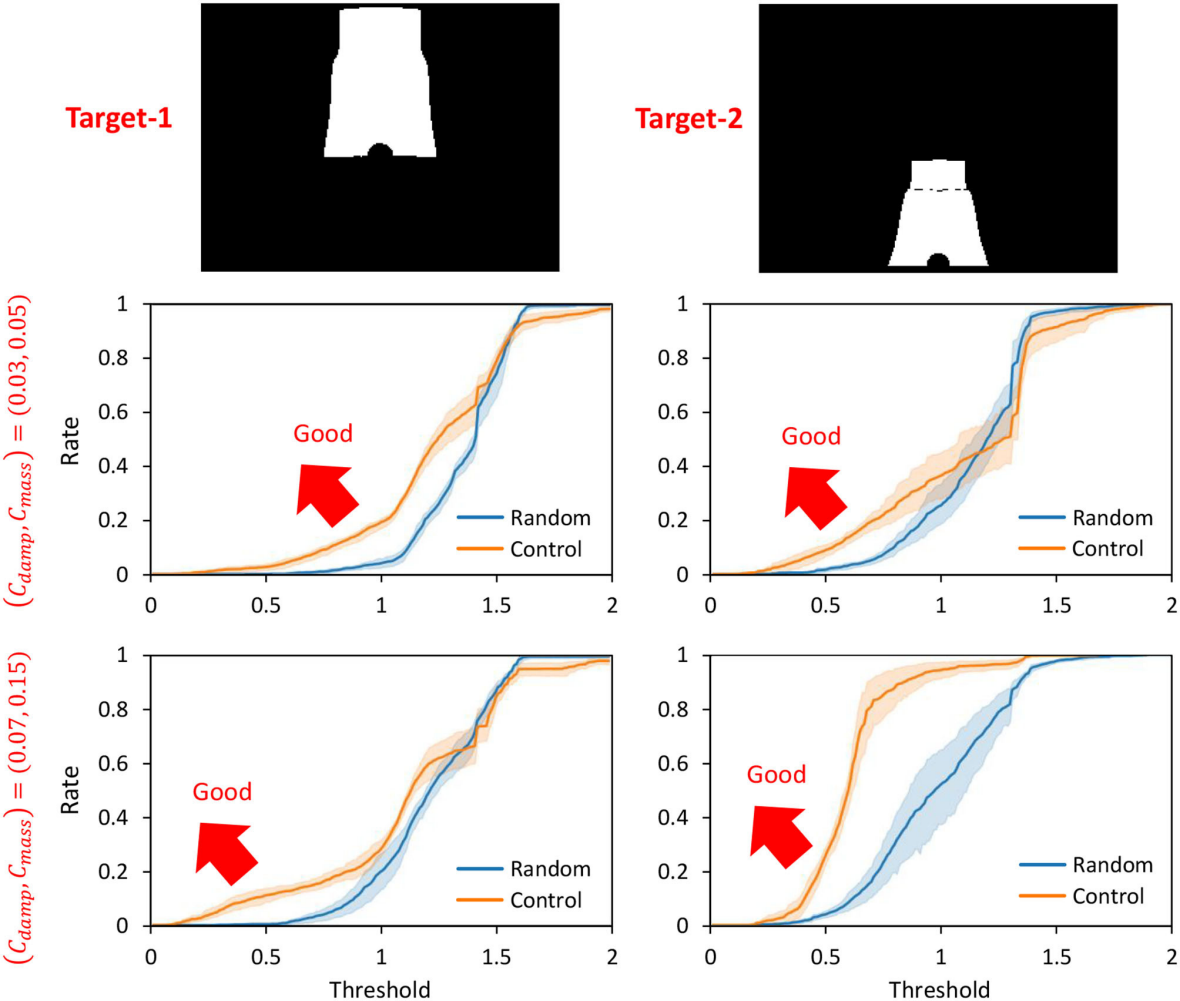
Simulation experiment: the rate (y-axis) of $||z^{ref}-z|{|}_{2}<$threshold (x-axis) when conducting experiments of dynamic manipulation of two cloths Target-1 and Target-2 when setting (*C*_*damp*_, *C*_*mass*_) = {(0.03, 0.05), (0.07, 0.15)} regarding Control and Random.

Next, we performed the control in Section 2.5 by setting the cloth characteristics to (*C*_*damp*_, *C*_*mass*_) = {(0.03, 0.05), (0.07, 0.15)} and by setting the current PB value to the PB obtained during the training of the object to be manipulated. Two images, Target-1 and Target-2 of Figure 6, are given as the target images of the cloth. We denote the case of control in this study as Control and the random trial as Random. For each of the two types of cloth, the Control and Random trials are performed for 50 s each, using the two target images. The percentage (y-axis) of $||z^{ref}-z|{|}_{2}$ lower than a certain threshold (x-axis) is shown in Figure 6. Figure 6 indicates that the more the graph expands in the upper-left direction (the larger the y-axis value becomes when the x-axis value is low), the more the target image is realized. Here, in order to verify the reproducibility, all experiments including learning and control are conducted five times, and the mean and variance of these trials are shown in the graphs. Note that the mean of $||z^{ref}-z|{|}_{2}$ in the initial condition shown in the left figure of Figure 4 is 1.55 for Target-1 and 1.36 for Target-2. In all cases, Control outperforms Random and succeeds in realizing the target image accurately. Since Target-1 is more difficult than Target-2, especially when (*C*_*damp*_, *C*_*mass*_) = (0.03, 0.05), the difference is clearly shown. Since the larger *C*_*damp*_ causes slower deformation of the cloth and makes it easier to lift up, the target image is relatively correctly realized when is set (*C*_*damp*_, *C*_*mass*_) to (0.07, 0.15), compared to when it is set to (0.03, 0.05). The reproducibility of performance with respect to learning and control is also high, especially in areas with a low threshold (x-axis), where the variance is small.

**Figure 7:**
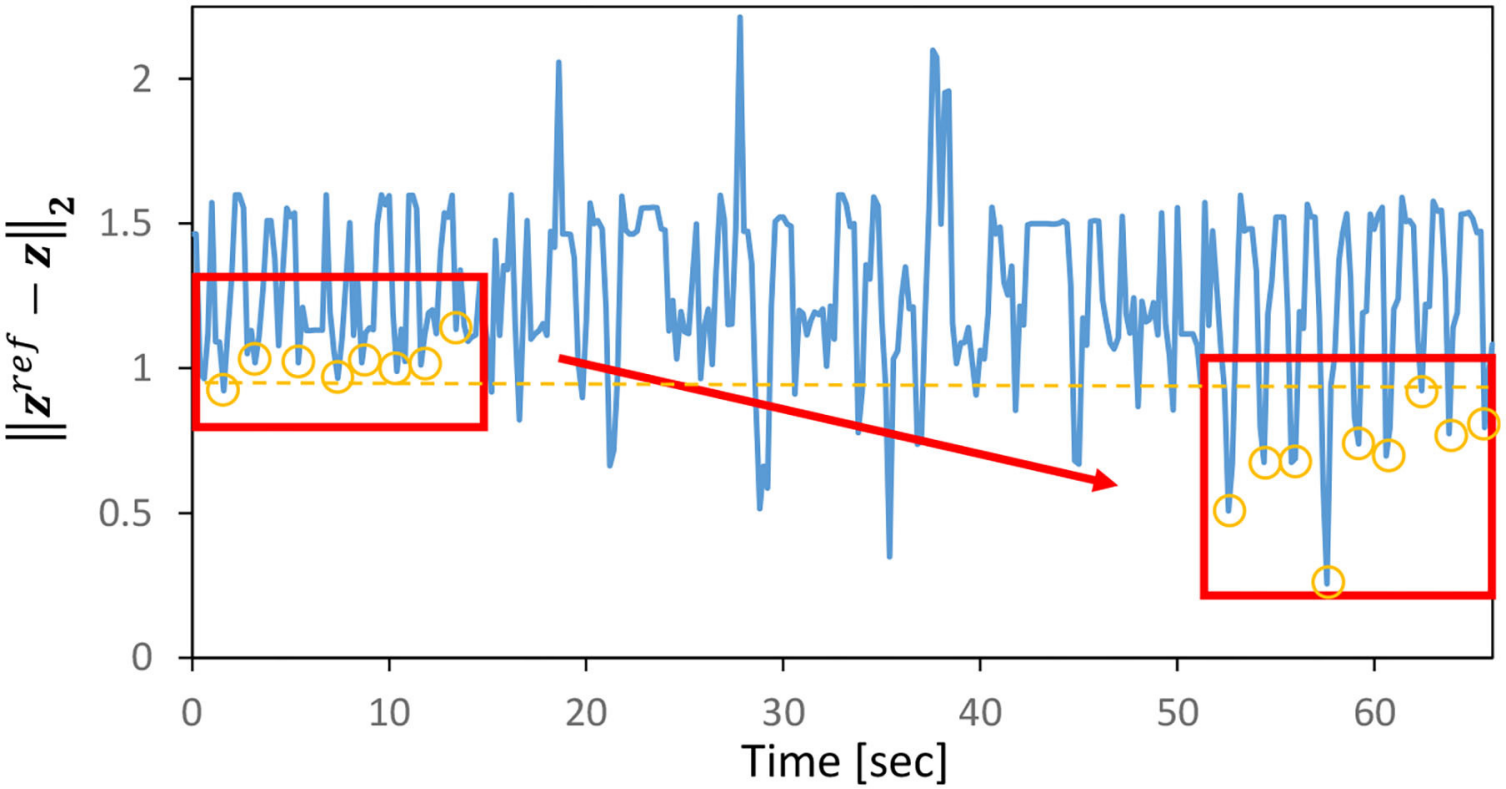
Simulation experiment: the transition of $||z^{ref}-z|{|}_{2}$ for the integrated experiment of online estimation and dynamic cloth manipulation.

Next, assuming that the current cloth properties are (*C*_*damp*_, *C*_*mass*_) = (0.03, 0.10) and the current PB values are (*C*_*damp*_, *C*_*mass*_) = (0.07, 0.15) obtained during training, we simultaneously perform the online material estimation in Section 2.4 and control in Section 2.5. Note that the target image is set as Target-1 of Figure 6. The transition of PB (integrated-traj) is shown in Figure 5, and the transition of $||z^{ref}-z|{|}_{2}$ is shown in Figure 7. It can be seen that the current PB gradually approaches the value obtained during training with (*C*_*damp*_, *C*_*mass*_) = (0.03, 0.10). Also, as PB becomes more accurate, $||z^{ref}-z|{|}_{2}$ periodically shows smaller values. In other words, the online material estimation and the learning control can be performed together, and the control becomes more accurate as PB becomes more accurate.

**Figure 8:**
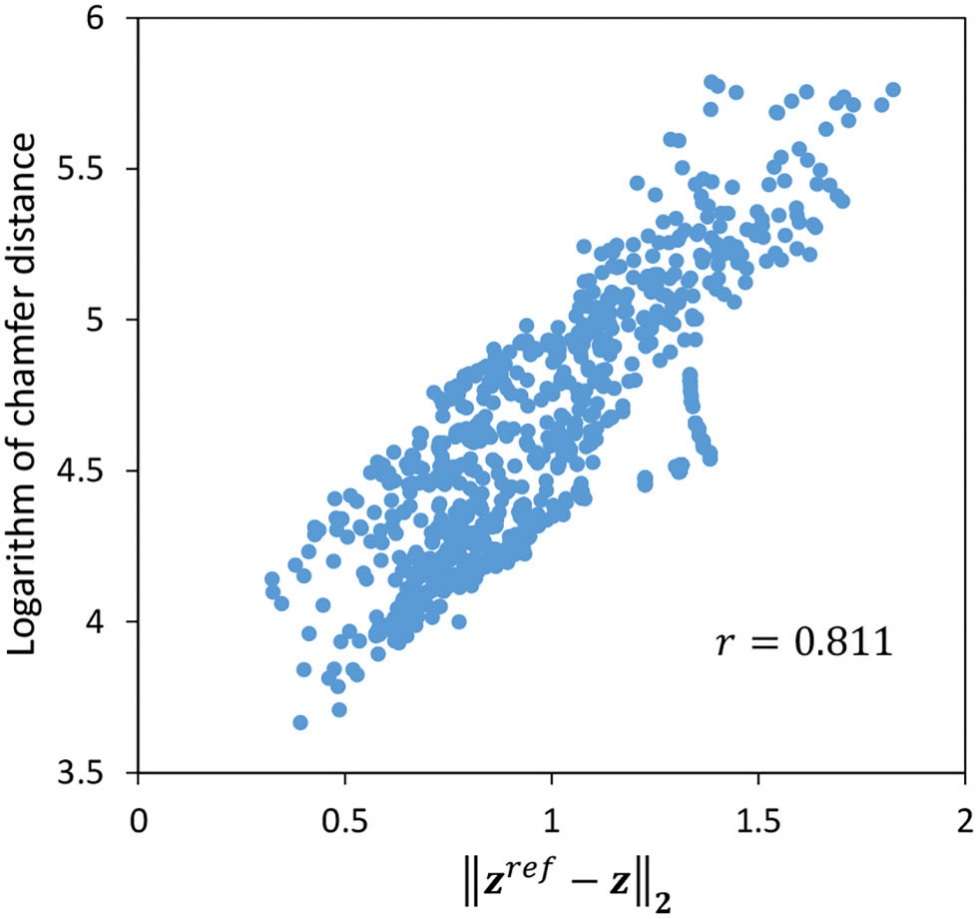
The correlation between the logarithm of chamfer distance and $||z^{ref}-z|{|}_{2}$.

Finally, we show that $||z^{ref}-z|{|}_{2}$ is close to the actual distance between the raw images since the control and its evaluation are basically performed with the error of the latent variable ***z*** in this study. We use Symmetric Chamfer Distance (Borgefors, 1988), which represents the similarity between binary images, to calculate the distance between the current raw image *I* and its target value *I*^*ref*^. The relationship between the logarithm of Chamfer Distance and $||z^{ref}-z|{|}_{2}$ when the target image is set to Target-2 and the cloth is randomly moved for 90 s is shown in Figure 8. The two values are well correlated, with a correlation coefficient of 0.811.

### 3.3. Actual Robot Experiment of Musashi-W

**Figure 9:**
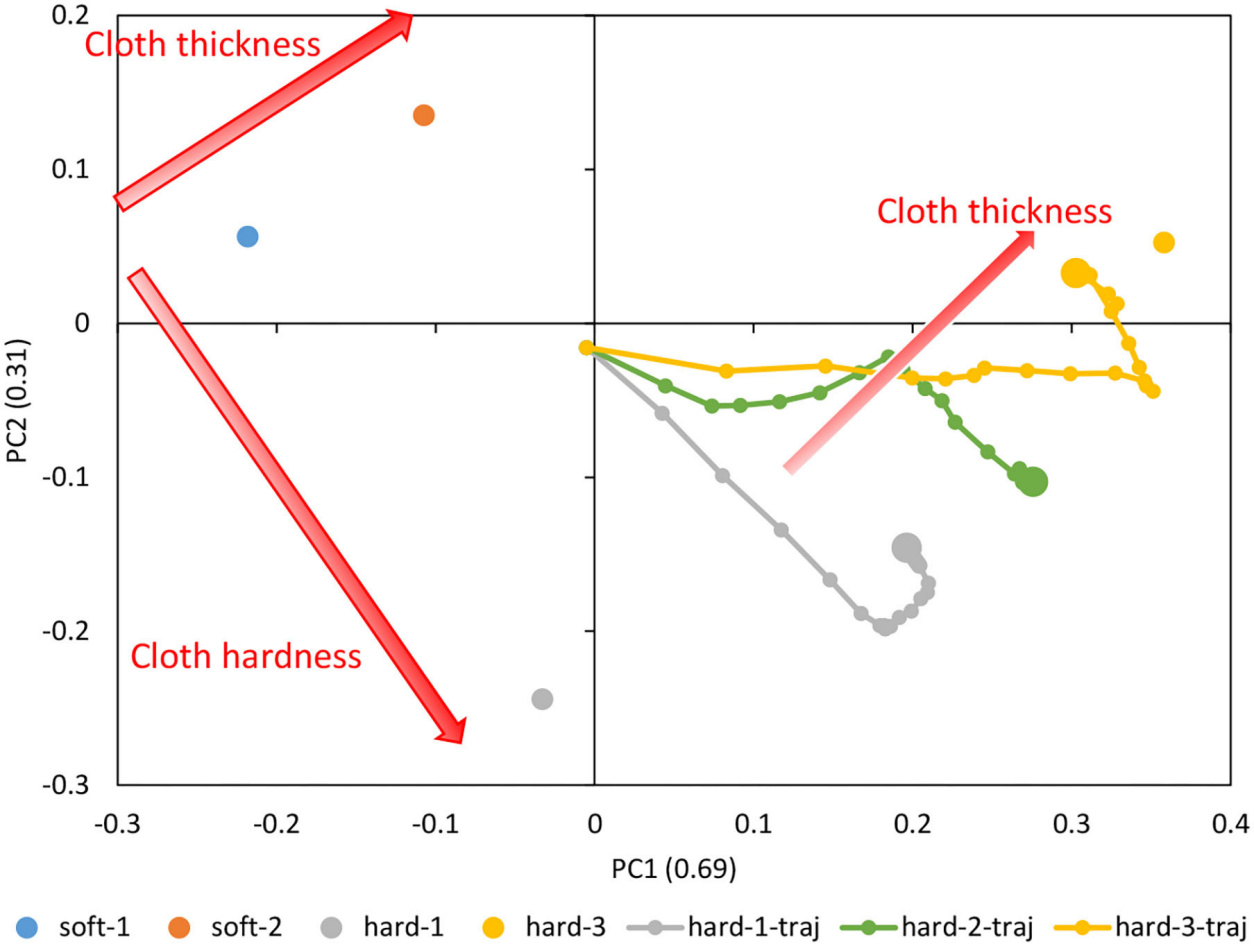
Actual robot experiment: the trained parametric bias for soft-1, soft-2, hard-1, and hard-3, and the trajectory of online updated parametric bias for hard-1, hard-2, and hard-3.

Motion data was collected by randomly commanding ***θ***^*ref*^ and ***k***^*ref*^ (Random) for about 80 s, and while human operation with GUI (joint angle is given by GUI, joint stiffness is randomly given) for about 80 s, for the cloths of soft-1, soft-2, hard-1, and hard-3, respectively. The random command ***k***^*ref*^ is set between [10, 70] [N]. Using a total of 3,000 data points, we trained DPMPB with the number of LSTM expansions being 20, the number of batches being 1,000, and the number of epochs being 600 (these parameters are empirically set). Principle Component Analysis (PCA) is applied to the parametric bias for each cloth obtained in the training, and its arrangement in the two-dimensional plane is shown in Figure 9. It can be seen that soft-1, soft-2, hard-1, and hard-3 are placed at the four corners, along with the thickness and stiffness of the cloth.

We execute the control in Section 2.5 and the online material estimation in Section 2.4 for about 30 s with each of the hard-1, hard-2, and hard-3 cloths. The trajectory of parametric bias is also shown in Figure 9. Note that the initial value of PB is **0** (the origin of PB is not necessarily at the origin of the figure, since PCA is applied). The updated PBs aligned in a straight line according to the thickness of the cloth. The updated PB of hard-3 is close to the PB of hard-3 obtained during training, while the updated PB of hard-1 deviated slightly from the PB of hard-1 obtained during training and was close to the PB of hard-3. The updated PB of hard-2, which is the data not used in the training, is located midway between hard-1 and hard-3. Therefore, we consider that the space of PB is self-organized according to the cloth thickness and cloth stiffness.

**Figure 10:**
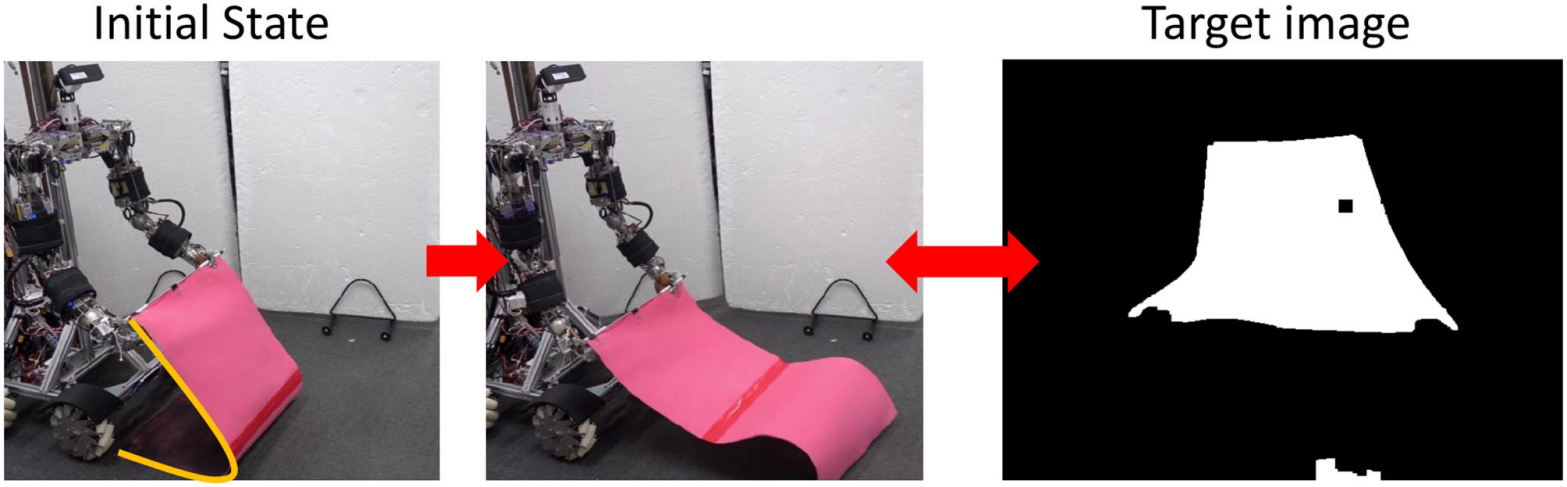
Experimental setup for the actual robot experiment: the initial state of cloth and target image of spread-out cloth.

**Figure 11:**
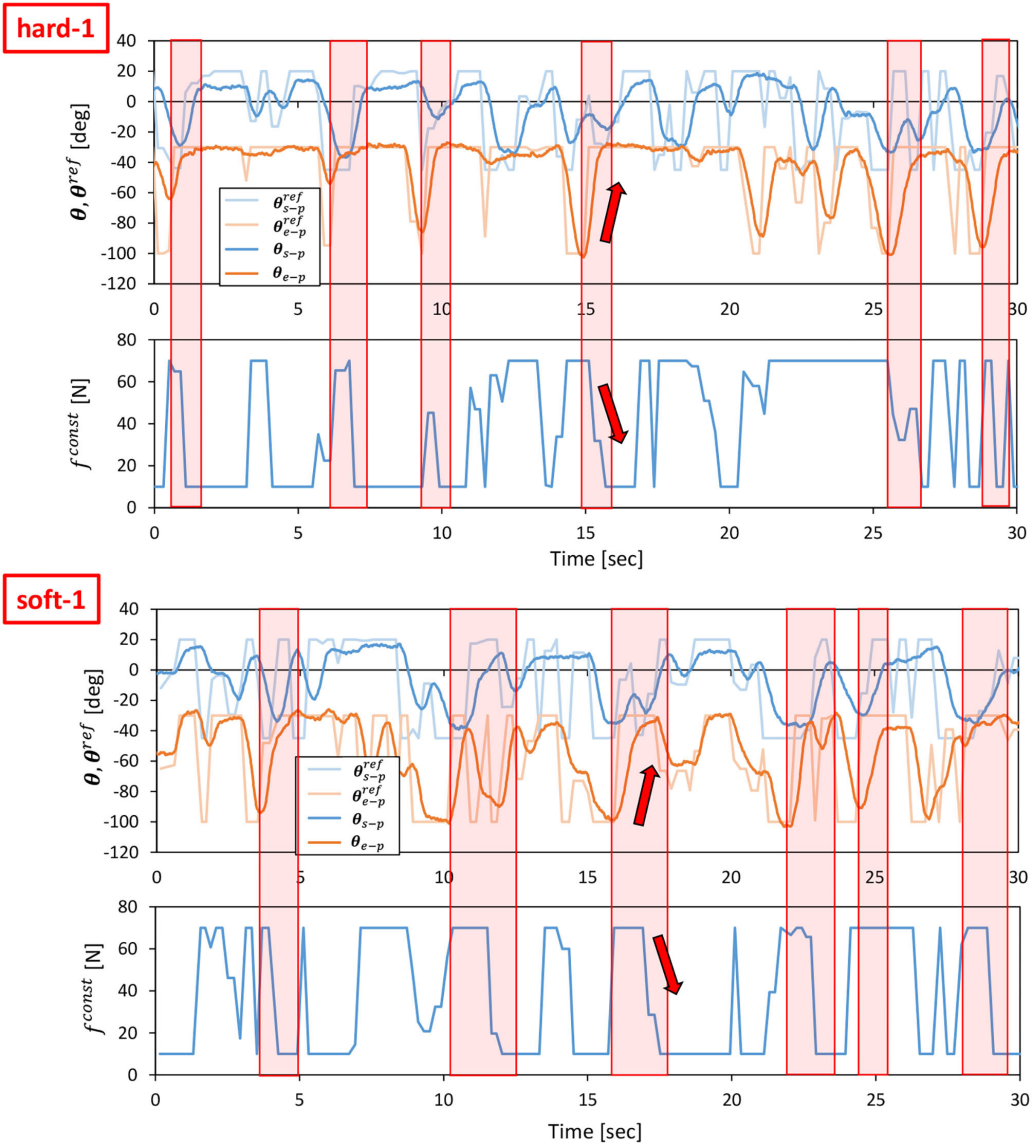
Actual robot experiment: examples of transitions of ***θ***^*ref*^, ***θ***, *f*^*const*^, and $||z^{ref}-z|{|}_{2}$ when conducting an experiment of dynamic manipulation of hard-1 **(upper graphs)** and soft-1 **(lower graphs)**.

**Figure 12:**
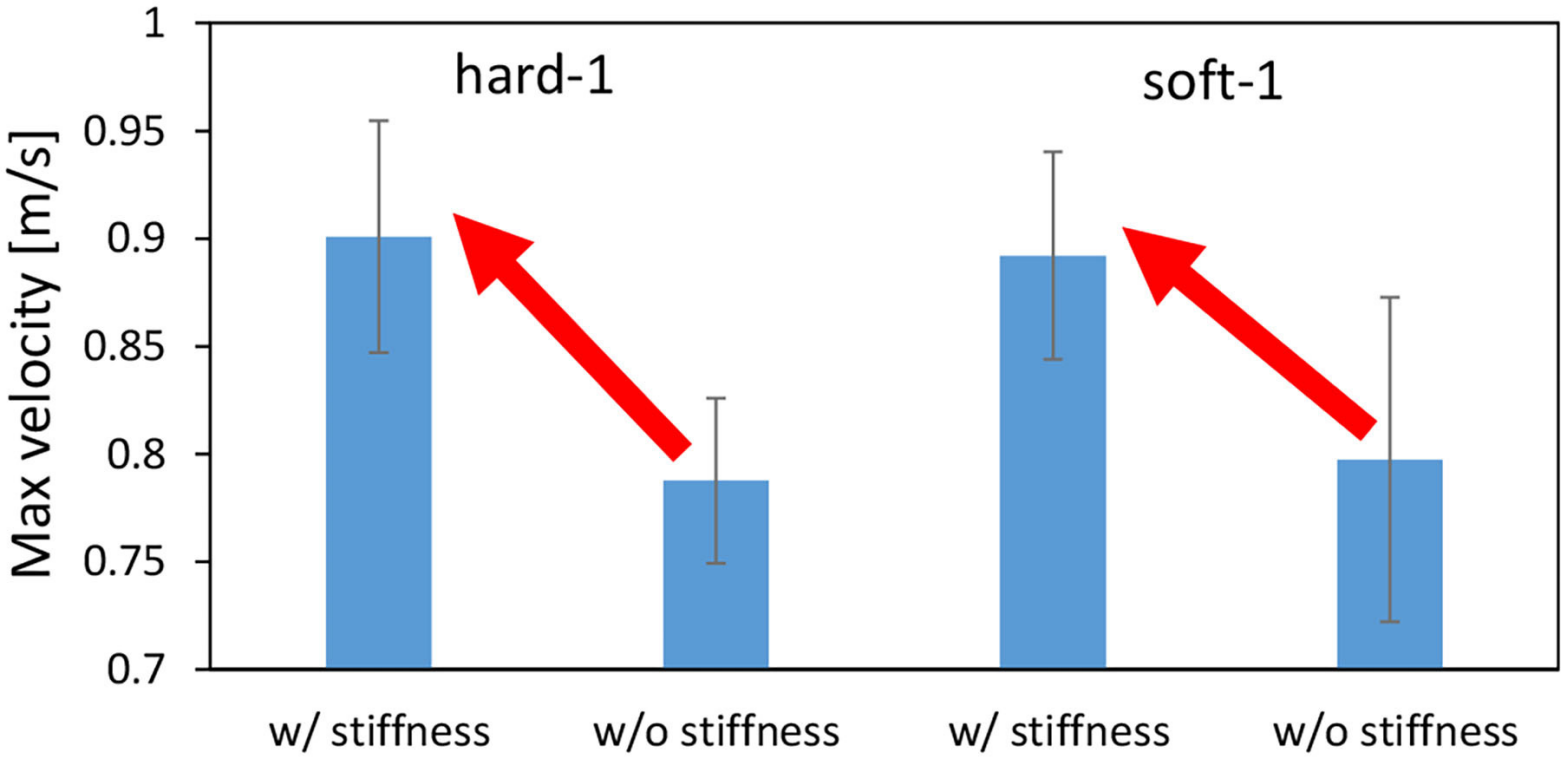
Actual robot experiment: the average and SD of the maximum velocity of the end effector when conducting experiments of dynamic manipulation of hard-1 and soft-1.

First, we performed the control in Section 2.5 with the value of PB set to the PB obtained during the training of the object to be manipulated. From the initial state of the cloth, as shown in the left figure of Figure 10, we use Section 2.5 to bring the cloth closer to the state of being spread out in the air as shown in the right figure of Figure 10. For hard-1 and soft-1, examples of transitions of the target joint angle ***θ***^*ref*^, the measured joint angle ***θ***, and the muscle tension *f*^*const*^ representing the target body stiffness are shown in Figure 11. Note that θ_*s*−*p*_ and θ_*e*−*p*_ represent the pitch joint angles of the shoulder and elbow, respectively. The area surrounded by the red frame is the main part of the spreading motion, where the upraised shoulder and elbow are lowered down significantly (the larger the joint angle is, the lower the arm is). Here, if we look at *f*^*const*^, we can see that in many cases, the stiffness is increased in the first half and decreased in the second half. This increases the hand speed and causes the cloth to spread out and float in midair, realizing the state shown in the right figure of Figure 10. The absolute values of the hand velocities when Equation (7) is not performed for ***k***^*ref*^ and *f*^*const*^ is fixed (= 10 N) (w/o stiffness), and when the control in Section 2.5 is performed (w/ stiffness), are shown in Figure 12. The figure shows the mean and variance of the maximum hand velocities during five 20-s experiments from the state shown in the left figure of Figure 10. It can be seen that the speed is about 12% faster when the change in the body stiffness is used. Also, looking at the difference between soft-1 and hard-1 in Figure 11, in hard-1, θ_*e*−*p*_ and θ_*s*−*p*_ move almost simultaneously and the process from raising to lowering the hand is quick. On the other hand, in soft-1, θ_*s*−*p*_ starts to move slightly later than θ_*e*−*p*_, and the process from raising to lowering the hand is often slow. The reason is that the soft cloth can maintain flight time even if the hand is lowered slowly at the end, while the hard cloth cannot maintain flight time unless the hand is lowered immediately. In this way, we can see that the way of manipulating the object varies depending on the difference in PB.

**Figure 13:**
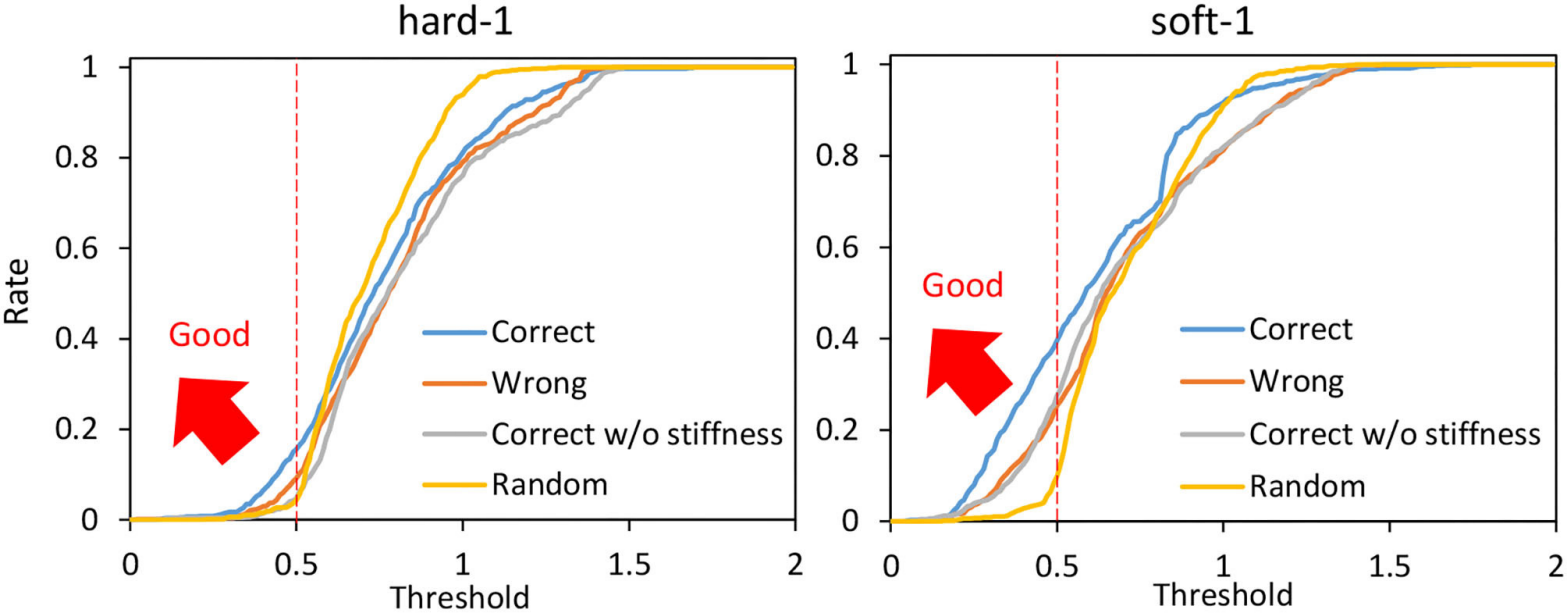
Actual robot experiment: the rate (y-axis) of $||z^{ref}-z|{|}_{2}<$threshold (x-axis) when conducting experiments of dynamic manipulation of hard-1 and soft-1 regarding Correct, Wrong, Correct without stiffness, and Random.

**Figure 14:**
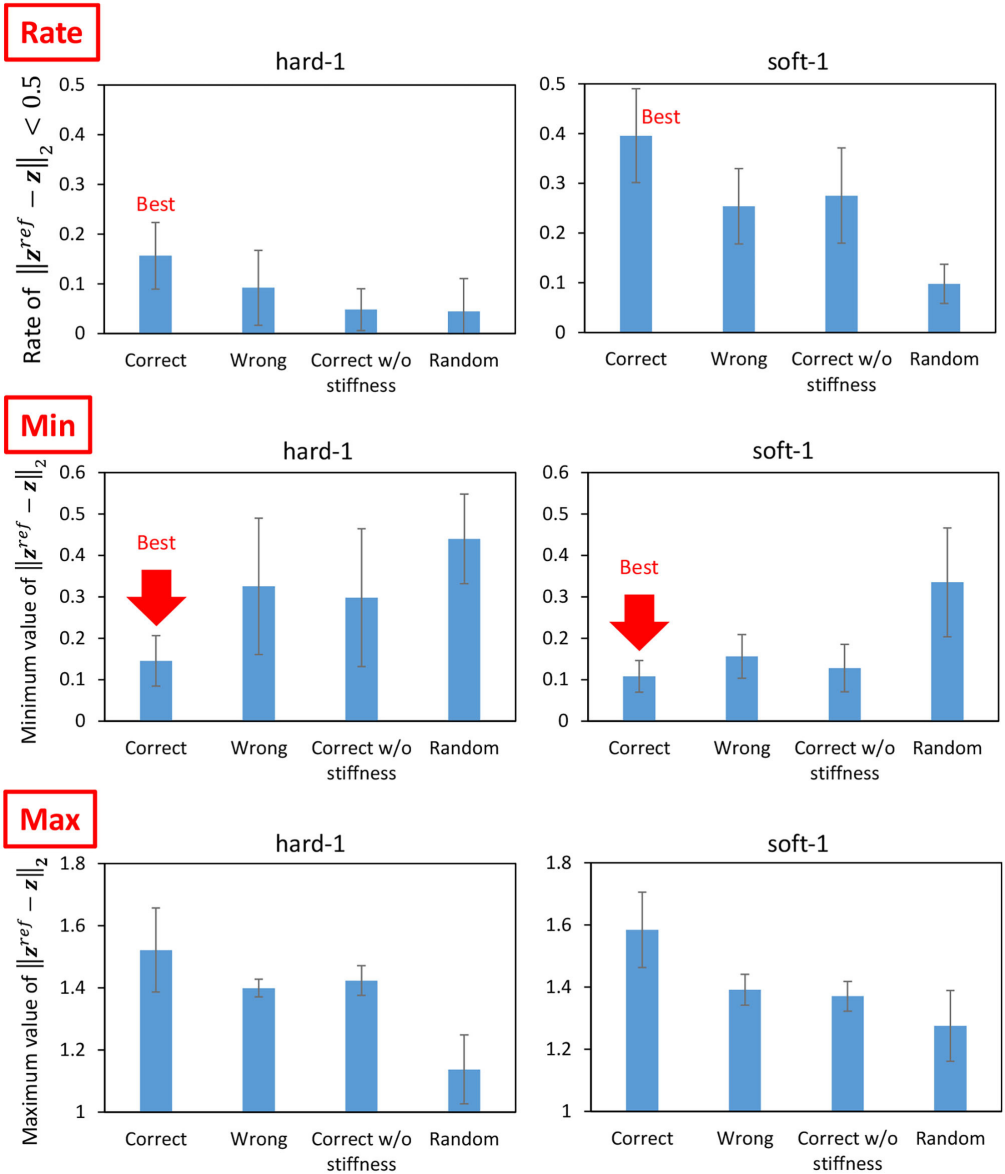
Actual robot experiment: the rate of $||z^{ref}-z|{|}_{2}<0.5$ **(upper graphs)**, the minimum value of $||z^{ref}-z|{|}_{2}$ **(middle graphs)**, and the maximum value of $||z^{ref}-z|{|}_{2}$ **(lower graphs)** when conducting experiments of dynamic manipulation of hard-1 and soft-1.

Next, we performed the cloth manipulation under several conditions and compared them. The trials with correct PBs (the PB of soft-1 is used for the manipulation of soft-1 and the PB of hard-1 is used for the manipulation of hard-1) are called Correct, the trials with wrong PBs (the PB of hard-1 is used for the manipulation of soft-1 and the PB of soft-1 is used for the manipulation of hard-1) are called Wrong, and random trials such as in Section 3.3 are called Random. The case where the PB is correct but Equation (7) is not performed for ***k***^*ref*^ and *f*^*const*^ is fixed (10N) is called Correct w/o stiffness. Under these four conditions, the hard-1 and soft-1 cloths are manipulated for 25 s five times from the state shown in the left graph of Figure 10. The average of the percentage (y-axis) with $||z^{ref}-z|{|}_{2}$ lower than a certain threshold (x-axis) is shown in Figure 13. The means and variances of the proportions of $||z^{ref}-z|{|}_{2}<0.5$ taken from Figure 13 are shown in the upper graphs of Figure 14, the means and variances of the minimum value of $||z^{ref}-z|{|}_{2}$ are shown in the middle graphs of Figure 14, and the means and variances of the maximum value of $||z^{ref}-z|{|}_{2}$ are shown in the lower graphs of Figure 14. Figure 13 indicates that the more the graph expands in the upper-left direction (the larger the y-axis value becomes when the x-axis value is low), the more the target image is realized. Note that the mean of $||z^{ref}-z|{|}_{2}$ for the initial condition shown in the left figure of Figure 10 is 0.86. For both hard-1 and soft-1, Correct is the best, and Random is the worst. Additionally, looking at the upper graphs of Figure 14, we can see the characteristics quantitatively. Although hard-1 is more difficult and, thus, has a lower realization rate in general, the results are consistent with the trend that Wrong and Correct w/o stiffness have lower realization rates than Correct. Looking at the middle graphs of Figure 14, the tendency of the minimum value of $||z^{ref}-z|{|}_{2}$ is also consistent with the upper graphs of Figure 14, and the lower the minimum value is, the higher the percentage of realization is. On the other hand, for the lower graphs of Figure 14, Correct is the highest, and Random is the lowest. This may indicate that the more accurate the method can realize the target image, the more likely it is to produce a state that is different from the target image since the state must be changed significantly in order to realize the target image.

### 3.4. Integrated Table Setting Experiment

**Figure 15:**
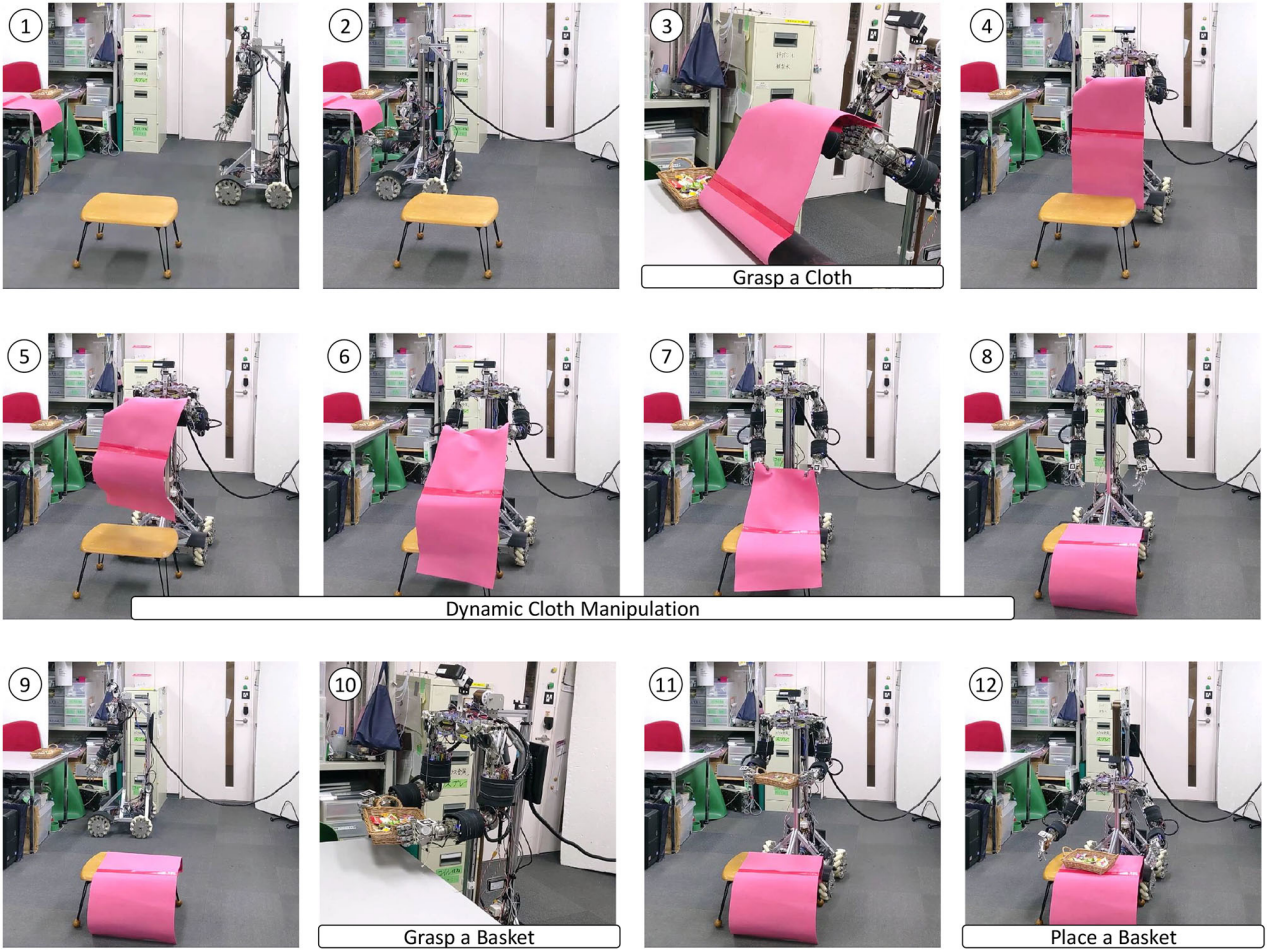
The integrated table setting experiment of grasping a cloth, manipulating it dynamically, and putting a basket on it.

We performed a series of motion experiments incorporating dynamic cloth manipulation. Musashi-W picked up hard-1 and laid it on the table like a table cloth by the proposed method, and placed a basket of sweets on it. The scene is shown in Figure 15. First, the robot recognizes the point cloud of the cloth and grasps the cloth between its thumb and index by visual feedback. Next, the robot spreads the cloth on the desk by dynamic cloth manipulation, which has been previously trained on a similar setup. Finally, the robot recognizes the point cloud of the basket and grasps the basket between both hands by visual feedback, and successfully places it on the table.

## 4. Discussion

We discuss the results obtained from the experiments in this study. First, simulation experiments show that the value of PB is self-organized according to the parameters of the cloth. The dynamics of the current cloth can be estimated online from the cloth manipulation data. On the other hand, we found that it is difficult to accurately estimate the parameters that do not contribute much to the change in dynamics. Through dynamic cloth manipulation experiments while changing the cloth parameters and the target image, our method can accurately realize the target image. By changing PB, the method can handle various cloth properties, and the target image can be given arbitrarily. The reproducibility of the performance on learning and control is also high. From the integrated experiment of online material estimation and dynamic cloth manipulation, we show that they can be performed simultaneously and that the closer the current PB is to the current cloth properties, the more accurately the target image can be realized.

From the actual robot experiments, we found that the value of PB is self-organized depending on the thickness and stiffness of clothes. As in the simulation experiments, the dynamics of the current cloth can be estimated online from the cloth manipulation data. We also found that the dynamics can be estimated at the correct point, which is the internally dividing point of the trained PBs, even for the data that is not available at the time of training. Through dynamic cloth manipulation experiments, we found that the behavior of the cloth manipulation varies depending on the PB which expresses the dynamics of the cloth. The joint angles and the speed were appropriately changed according to the material of the cloth to be manipulated. In addition, the stiffness value affects the speed of the hand movements, and when the stiffness value is optimized, the hand speed is increased by about 12% compared to the case without its optimization. When PB does not match the current cloth or the stiffness value is not optimized, the performance decreased compared to the case where PB is correct and the variable stiffness is used. In addition, we found that in order to realize the target image correctly, it is necessary to go through a state that is far from the target image. From these results, we found that the dynamics of the cloth material are embedded and estimated online through parametric bias, that variable stiffness control can be used explicitly to improve the hand speed, and that the target cloth image can be realized more accurately by using deep predictive model learning and backpropagation technique to the control command.

While our method can greatly improve the ability of dynamic cloth manipulation of robots, there are still some issues to be solved. First, in this study, the body stiffness is substituted by a certain single value, but in humans, the body stiffness can be set more flexibly. It is necessary to discuss the degree of freedom of the body stiffness in the future. Next, we consider that it is difficult for our method to deal with the case of large nonlinear changes such as the cloth leaving the hand or changing the grasping position. There is no method that can handle such highly nonlinear, discontinuous, and high-dimensional states in trials using only actual robots, and this is an issue for the future. Finally, we are still far from reaching human-like adaptive dynamic cloth manipulation. Humans are capable of stretching and spreading the cloth by focusing on small wrinkles, instead of just looking at the entire cloth universally. In addition, since the current hardware is not as agile and soft as humans, it will be necessary to develop both hardware and software.

## 5. Conclusion

In this study, we developed a deep predictive model learning method that incorporates quick manipulation using variable stiffness mechanisms, and response to changes in cloth material for more human-like dynamic cloth manipulation. For variable stiffness, the command value is calculated by the backpropagation technique using the joint angle and the body stiffness value as control commands. For the adaptation to change in cloth material, we embed information about the cloth dynamics into parametric bias. The dynamics of the cloth material can be estimated online even when it is not included in the training data, and it is shown that the characteristics of the dynamic cloth manipulation vary greatly depending on the material. In addition, when the stiffness value is appropriately set according to the motion phase, the speed can be increased by about 12% compared to the case without variable stiffness control, and the target cloth state can be realized more accurately. In the future, we would like to develop a method to handle more complicated cloth manipulation tasks in a unified manner.

## Data Availability Statement

The raw data supporting the conclusions of this article will be made available by the authors, without undue reservation.

## Author Contributions

KK contributed to conception and design of this study, implemented the software, conducted the experiments, and wrote this manuscript. AM and MB conducted the experiments with KK. KO and MI supervised this project and contributed to conception of this study. All authors contributed to the article and approved the submitted version.

## Funding

This research was partially supported by JST ACT-X grant no. JPMJAX20A5 and JSPS KAKENHI grant no. JP19J21672.

## Conflict of Interest

The authors declare that the research was conducted in the absence of any commercial or financial relationships that could be construed as a potential conflict of interest.

## Publisher's Note

All claims expressed in this article are solely those of the authors and do not necessarily represent those of their affiliated organizations, or those of the publisher, the editors and the reviewers. Any product that may be evaluated in this article, or claim that may be made by its manufacturer, is not guaranteed or endorsed by the publisher.

## Supplementary Material

Video 1 Attached is a video of experiments.
